# Fragile X–Related Protein 1 Regulates Nucleoporin Localization in a Cell Cycle–Dependent Manner

**DOI:** 10.3389/fcell.2021.755847

**Published:** 2021-12-16

**Authors:** Arantxa Agote-Arán, Junyan Lin, Izabela Sumara

**Affiliations:** ^1^ Institut de Génétique et de Biologie Moléculaire et Cellulaire (IGBMC), Illkirch, France; ^2^ Centre National de la Recherche Scientifique UMR 7104, Strasbourg, France; ^3^ Institut National de la Santé et de la Recherche Médicale U964, Strasbourg, France; ^4^ Université de Strasbourg, Strasbourg, France

**Keywords:** cell cycle, G1 phase, nucleoporins, nuclear pore complex, phase separation, FXR1

## Abstract

Nuclear pore complexes (NPCs) are embedded in the nuclear envelope (NE) where they ensure the transport of macromolecules between the nucleus and the cytoplasm. NPCs are built from nucleoporins (Nups) through a sequential assembly order taking place at two different stages during the cell cycle of mammalian cells: at the end of mitosis and during interphase. In addition, fragile X–related proteins (FXRPs) can interact with several cytoplasmic Nups and facilitate their localization to the NE during interphase likely through a microtubule-dependent mechanism. In the absence of FXRPs or microtubule-based transport, Nups aberrantly localize to the cytoplasm forming the so-called cytoplasmic nucleoporin granules (CNGs), compromising NPCs’ function on protein export. However, it remains unknown if Nup synthesis or degradation mechanisms are linked to the FXRP–Nup pathway and if and how the action of FXRPs on Nups is coordinated with the cell cycle progression. Here, we show that Nup localization defects observed in the absence of FXR1 are independent of active protein translation. CNGs are cleared in an autophagy- and proteasome-independent manner, and their presence is restricted to the early G1 phase of the cell cycle. Our results thus suggest that a pool of cytoplasmic Nups exists that contributes to the NPC assembly specifically during early G1 to ensure NPC homeostasis at a short transition from mitosis to the onset of interphase.

## Introduction

In eukaryotic cells, the nuclear envelope (NE) separates the nucleus from the cytoplasm, and the communication between these two compartments is crucial for cell viability. Nuclear pore complexes (NPCs) are large protein assemblies that constitute the transport channels and regulate the exchange of macromolecules through the NE. In mammalian cells, about 30 different nucleoporins (Nups), present in a well-defined number of copies, build subcomplexes that assemble together into eight-fold symmetrical NPCs of 120 MDa (Knockenhauer and Schwartz, 2016). In higher eukaryotes, which undergo open mitosis, there are two main NPC assembly pathways operating during different stages of the cell cycle. At the end of mitosis, previously disassembled NPCs from the the mother cell rapidly reform, concomitant with NE reassembly following the so-called postmitotic NPC assembly pathway. During interphase, as cells grow, new NPCs need to be inserted into an enclosed NE, following the so-called interphasic NPC assembly pathway. This pathway is especially active in early G1 phase where a burst of NPC biogenesis has been observed immediately after NE reformation (Dultz and Ellenberg, 2010; Rampello et al., 2020). The postmitotic and interphasic pathways are based on fundamentally distinct mechanisms probably due to the different conditions in which they function (the presence or absence of pre-existing building blocks and open or enclosed NE) (reviewed by Weberruss and Antonin, 2016, Otsuka and Ellenberg, 2018). In the postmitotic pathway, ELYS initiates the NPC assembly on segregated chromosomes, while during interphase, both Nup153 and POM121 can drive the *de novo* assembly of NPCs into an enclosed NE (D’Angelo et al., 2006; Doucet et al., 2010; Vollmer et al., 2015). The Nups building the so-called Y-complex are also critical for NPC assembly, both after mitosis and during interphase (Walther et al., 2003; Doucet et al., 2010).

A third pathway has been described in cells with rapid cell cycles, such as germ, early embryonic, and cancer cells. It is based on the existence of cytoplasmic stacks of double membranes, termed annulate lamellae (AL), which accommodate a high number of preformed NPCs that can be inserted *en bloc* into the expanding NE (Hampoelz et al., 2016). Recently, Ren et al. suggested that rather than being a cell type–specific pathway, the AL-based NPC assembly is an intermediate step in the postmitotic pathway in higher eukaryotic cells (Ren et al., 2019).

Among other factors, the cohesive abilities of several Nups contribute to the NPC assembly (Onischenko et al., 2017). In fact, one-third of the Nups contain several copies of phenylalanine–glycine (FG) repeats (so-called FG-Nups) which are intrinsically disordered and engage in multivalent protein–protein interactions. Due to these characteristics, FG-Nups have the tendency to undergo liquid–liquid phase separation (LLPS), and examples of Nup condensates under physiological conditions were previously reported. For instance, FG-Nups in the central channel of the NPC form a sieve-like hydrogel that constitutes the permeability barrier and is essential for cell viability (Schmidt and Görlich, 2016). Nup condensates have also been suggested to constitute the precursors and assembly platforms for AL during *Drosophila* oogenesis (Hampoelz et al., 2019). Interestingly, mRNAs from several Nups were found to be localized to the surface of AL, but this enrichment was lost upon translation inhibition, suggesting that translation and condensation of Nups can take place in these compartments (Hampoelz et al., 2019). On the other hand, several examples of aberrant condensation of Nups were observed, such as in the pathological protein inclusions present in neurodegenerative diseases (Li and Lagier-Tourenne, 2018; Hutten and Dormann, 2019) and in stress granules (Zhang et al., 2018). Altogether, these studies suggest that LLPS of Nups may occur in different cellular compartments, both under physiological and pathological conditions.

Recently, the family of fragile X–related proteins (FXRPs) (FXR1 and FXR2, and fragile X mental retardation protein (FMRP)) was implicated in the regulation of Nup localization (Agote-Aran et al., 2020). FXR1 can interact with several Nups and drive their localization to the NE during early interphase likely through a dynein- and microtubule-dependent mechanism. In the absence of FXRPs or microtubule-based transport, Nups aberrantly localize to the cytoplasm, forming the so-called cytoplasmic nucleoporin granules (CNGs) transiently compromising NPCs’ protein export function at the NE. However, since FXRPs are RNA-binding proteins playing several crucial roles in protein translation (Li and Zhao, 2014), it is important to understand if Nup synthesis is linked to the FXRP–Nup pathway. Likewise, it remains to be understood what is the exact fate of CNGs during cell cycle progression and if their dynamics involve any known degradation mechanisms.

Here, we show that the formation of CNGs in early G1 phase is independent of active protein translation. The presence of Nup granules induced by the downregulation of FXR1 is restricted to the early G1 phase, and the CNGs can no longer be observed after G1/S transition. Furthermore, their disappearance is independent of autophagy or proteasomal degradation pathways. We propose a model where a pool of nucleoporins synthesized in the previous cell cycle remains in the cytoplasm after the postmitotic NPC assembly. This Nup pool has to be incorporated into the growing nucleus in early G1 phase to ensure NPC homeostasis at a short transition from mitosis to the onset of interphase.

## Results

### Active Protein Translation Is Not Required for the Formation of the Cytoplasmic Nucleoporin Granules

mRNAs from several Nups and importins were found to be enriched on the surface of AL in *Drosophila* oocytes. The mRNA localization was lost upon translation inhibition, suggesting active translation of Nups in these compartments. Furthermore, the authors suggested that Nup translation may contribute to their effective condensation into granules formed prior to the AL assembly (Hampoelz et al., 2019). Although no polyadenylated mRNA enrichment was detected at the CNGs previously (Agote-Aran et al., 2020), it cannot be formally excluded that the expression of specific Nups is regulated by FXRPs. Moreover, given the established roles of FXRPs in translation mechanisms, these proteins could not only transport Nups to the NE (Agote-Aran et al., 2020) but also regulate the translation of Nups during interphase.

First, we aimed to validate HeLa cells as an appropriate cellular model to study the dynamics of CNGs. Similar to HeLa cells, about 20% of non-transformed RPE-1 (Supplementary Figures S1A, B) and chromosomally stable diploid DLD-1 colon carcinoma cells (Supplementary Figures S1C, D) showed the presence of CNGs, which was strongly increased upon the deletion of FXR1 (Supplementary Figures S1A–D). These results are in accordance with the previously published findings in human fibroblasts, human-induced pluripotent stem cells (iPSCs), and mouse embryonic fibroblasts (MEFs) (Agote-Aran et al., 2020), and validate the use of HeLa cells for further experiments.

**FIGURE 1:**
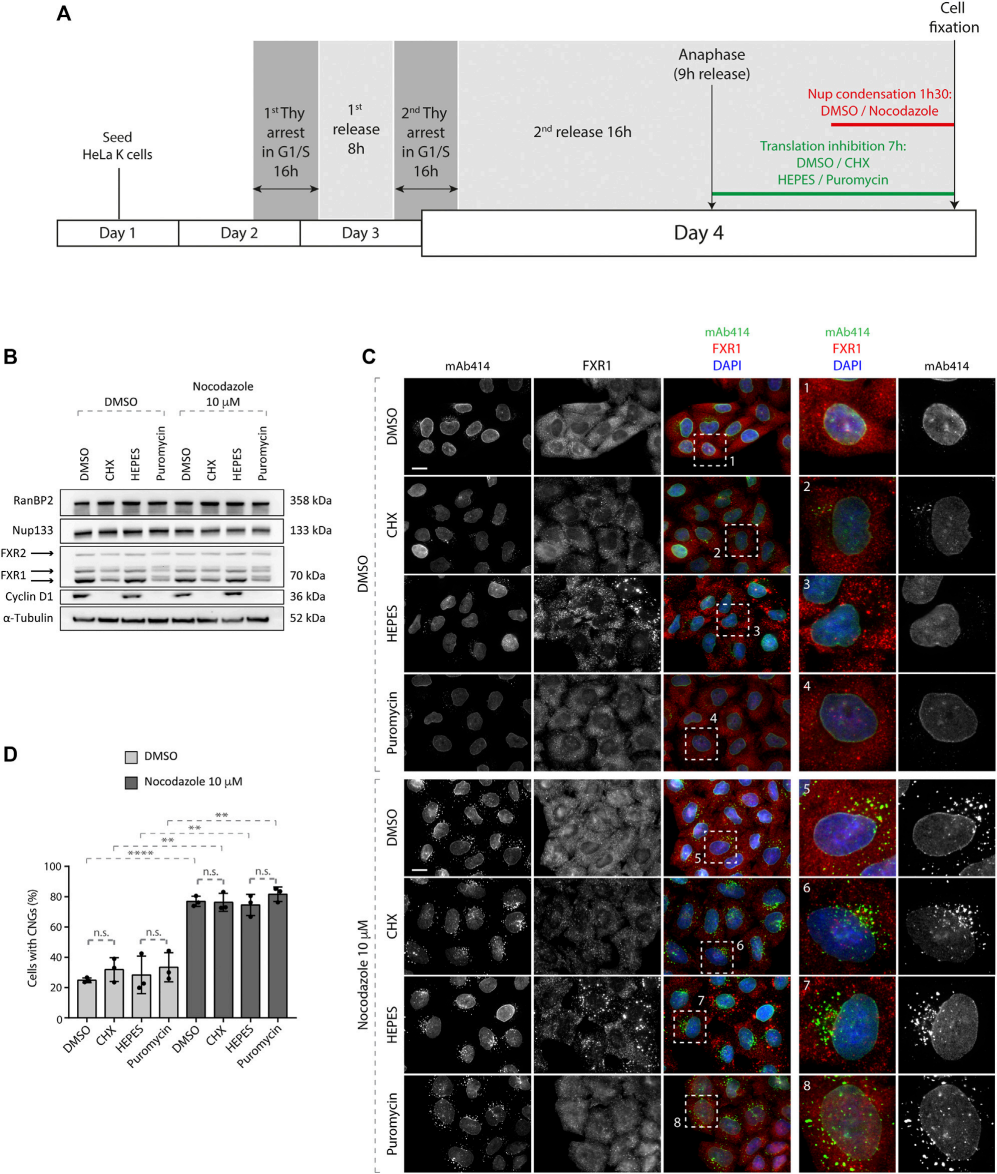
Nup granule formation upon microtubule depolymerization does not depend on active translation. **(A)** Scheme of the experimental setup. HeLa K cells were seeded on coverslips, synchronized by double thymidine block, and released from G1/S arrest. Nine hours after release (when synchronized cells were in anaphase), the cells were incubated with translational inhibitors (100 μg/ml cycloheximide (CHX) or 200 μg/ml puromycin) or the vehicles (DMSO and HEPES, respectively) for additional 7 h. During the last 90 min, Nup granules were induced by the addition of 10 µM nocodazole or the vehicle (DMSO) in the presence of the translational inhibitors. Subsequently, the cells were analyzed by Western blotting **(B)** and immunofluorescence microscopy **(C, D)**. **(B)** Whole cell lysates from cells treated as in **(A)** were analyzed by Western blotting. **(C, D)** Cells treated as in **(A)** were analyzed by immunofluorescence microscopy **(C)** and the percentage of cells with cytoplasmic nucleoporin granules (CNGs) was quantified. **(D)** 5,900 cells were analyzed (mean ± SD, **p < 0.01; ****p < 0.0001, n.s. = non-significant, N = 3). The magnified framed regions are shown in the corresponding numbered panels. Data information: scale bars are 5 μm. Statistical significance was assessed by one-way ANOVA test with Sidak’s correction.

To test if the formation of CNGs requires active protein translation in HeLa cells, we designed the following experimental setup (Figure 1A); HeLa cells synchronized by double thymidine block and release (DTBR) were treated with the inhibitors of translation, namely, cycloheximide (CHX) and puromycin, at the 9-h time point after the second thymidine release, that is, at the time when the synchronized cells reached anaphase and no more cyclin B needed to be produced for mitosis to proceed. The inhibitory effect of CHX and puromycin on active translation was confirmed by Western blotting against FXR1 and cyclin D1, where protein levels of both markers were decreased relative to the solvent-treated cells (Figure 1B). No effects on the protein levels of RanBP2 and Nup133 were observed under all conditions. Since nocodazole treatment leads to rapid CNG formation (Agote-Aran et al., 2020), it was used for the last 1.5 h in the experimental protocol (Figure 1A). As expected, microtubule depolymerization by nocodazole led to strong induction of CNGs labeled by the mAb414 antibody, which recognizes a panel of FG-Nups (Figures 1C,D). Treatment with CHX and puromycin did not affect the percentage of cells with CNGs upon microtubule depolymerization (Figures 1C,D) or CNGs’ appearance. Inhibition of protein translation did not induce Nup granules in cells untreated with nocodazole (Figures 1C,D). Based on the strong reduction in the protein levels of the G1 marker cyclin D1 upon translation inhibition but no changes in the protein levels of RanBP2 and Nup133 (Figure 1B), we presume that no new Nups were synthesized under these conditions. Yet, CNGs could be induced by microtubule depolymerization, suggesting that the ongoing translation is not required for their formation.

### CNGs Are Not Cleared by the Proteasome or Autophagy

**FIGURE 2:**
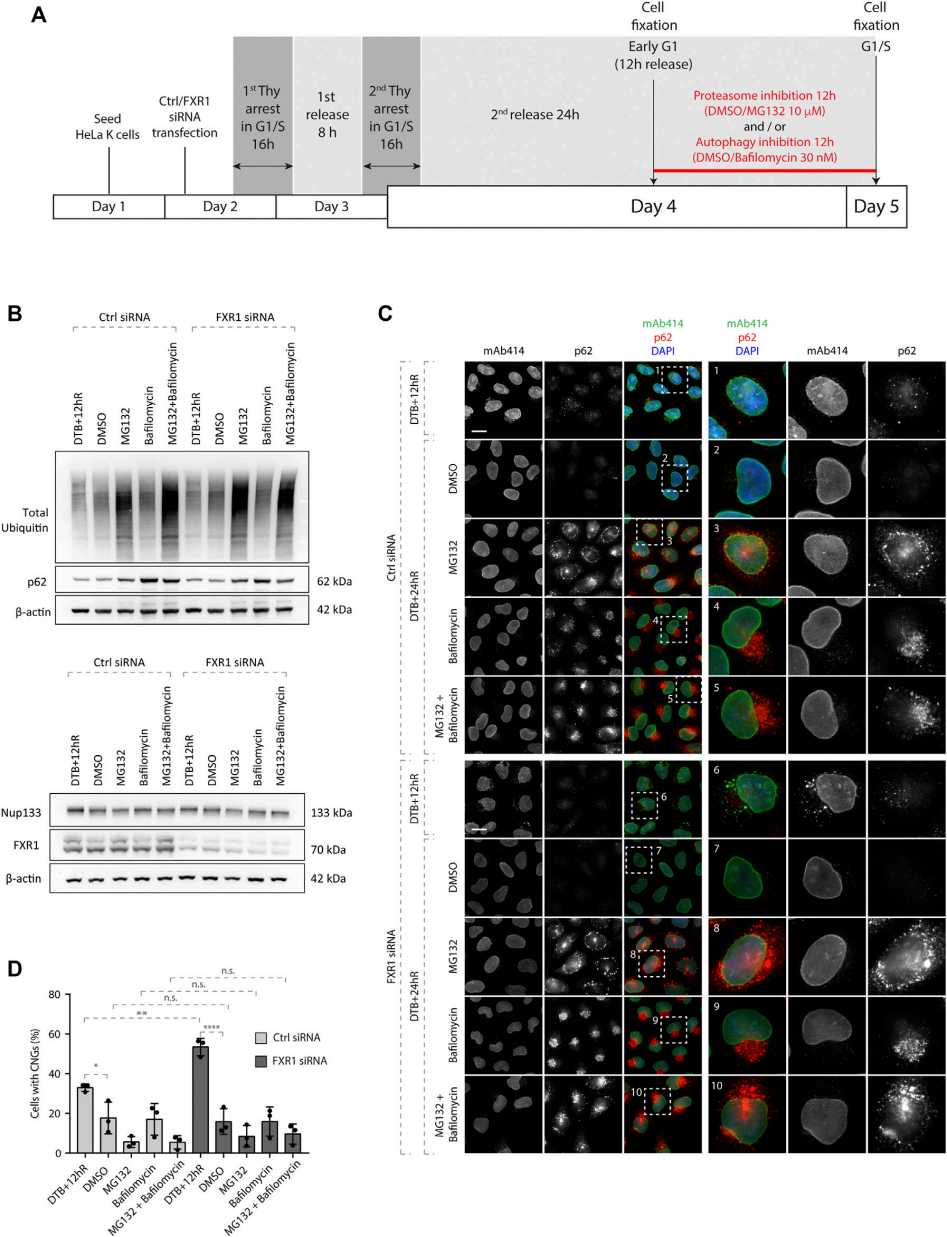
CNGs induced by FXR1 downregulation are not cleared through proteasome or autophagy. **(A)** Scheme of the experimental setup. HeLa K cells were seeded on coverslips, treated with the indicated siRNAs, synchronized by double thymidine block, and released from G1/S arrest. Twelve hours after release (when synchronized cells were in early G1), the cells were incubated with the proteasome inhibitor (10 µM MG132) and/or autophagy inhibitor (30 nM bafilomycin) or the vehicle (DMSO) for additional 12 h. During the last 90 min, Nup granules were induced by the addition of nocodazole or the vehicle (DMSO) in the presence of translational inhibitors. Subsequently, the cells were analyzed by Western blotting **(B)** and immunofluorescence microscopy **(C, D)**. **(B)** Whole cell lysates from cells treated as in **(A)** were analyzed by Western blotting. **(C, D)** Cells treated as in **(A)** were analyzed by immunofluorescence microscopy (**C**) and the percentage of cells with cytoplasmic nucleoporin granules (CNGs) was quantified. **(D)** 7,100 cells were analyzed (mean ± SD, *p < 0.05; **p < 0.01; and ****p < 0.0001; n.s. = non-significant, N = 3). The magnified framed regions are shown in the corresponding numbered panels. Data information: scale bars are 5 μm. Statistical significance was assessed by one-way ANOVA test with Sidak’s correction.

NPCs embedded in double-membrane vesicles were observed and proposed to be sequestered in autophagosomes, and NPC turnover involves the core autophagy machinery in *S. cerevisiae* (Lee et al., 2020; Tomioka et al., 2020). In addition, Nups were shown to be degraded by both autophagy and the proteasome (Webster et al., 2014; Lee et al., 2020; Tomioka et al., 2020). We asked if CNGs persist or can be cleared as cells progress to the next cell cycle, and if the autophagy or proteasome-based degradation pathways are involved in the turnover of the CNGs. To test this, we induced CNGs in a nocodazole-independent manner due to the fact that several studies demonstrated an essential role of microtubules in autophagosome formation (Mackeh et al., 2013) and used the siRNA specific to FXR1 which was previously shown to induce CNGs (Agote-Aran et al., 2020). The cells were synchronized in the early G1 phase as shown in Figure 1A, and MG132 (proteasome inhibitor), bafilomycin (autophagy inhibitor), or both agents were simultaneously used for the following 12 h as the cells reached the next G1/S stage. As a control and to monitor the turnover of CNGs under normal conditions, we treated the cells with the solvent (DMSO) (Figure 2A). As expected, proteasome inhibition by MG132 increased the levels of ubiquitin and ubiquitylated proteins relative to treatment with DMSO or bafilomycin, and the autophagy marker p62 accumulated upon both bafilomycin and MG132 treatments (Figures 2B,C) relative to the DMSO control. Likewise, treatment with FXR1-specific siRNA decreased the proteins levels of different FXR1 isoforms, and importantly, none of the treatments affected the protein levels of Nup133 (Figure 2B). Interestingly, CNGs induced by FXR1 downregulation at early G1 (12 h after the release from the double thymidine block) did not persist until the following G1/S stage (24 h after the release), and the inhibition of proteasome, autophagy, or both did not affect the disappearance of CNGs (Figures 2C,D). Inhibition of proteasome and/or autophagy also did not lead to the accumulation of Nup granules in the control siRNA conditions (Figures 2C,D), while as expected, the autophagy marker p62 formed aggregates in cells treated with bafilomycin and/or MG132 in control- and FXR1 siRNA–treated cells (Figure 2C). In line with these results, we were not able to observe co-localization between CNGs and the lysosomal marker Lamp1, upon solvent treatment or lysosomal hydrolase inhibition with pepstatin A and E64d in FXR1-deficient cells (Supplementary Figures S2A,B), suggesting that lysosomes are not the final destination compartment of CNGs. These results suggest that CNGs induced by FXR1 downregulation can be cleared as cells progress to the next cell cycle in a proteasome- and autophagy-independent manner and that regulation of Nup localization by FXR1 might be cell cycle dependent.

### Regulation of Nucleoporin Localization by FXR1 Occurs in a Cell Cycle–specific Manner

**FIGURE 3:**
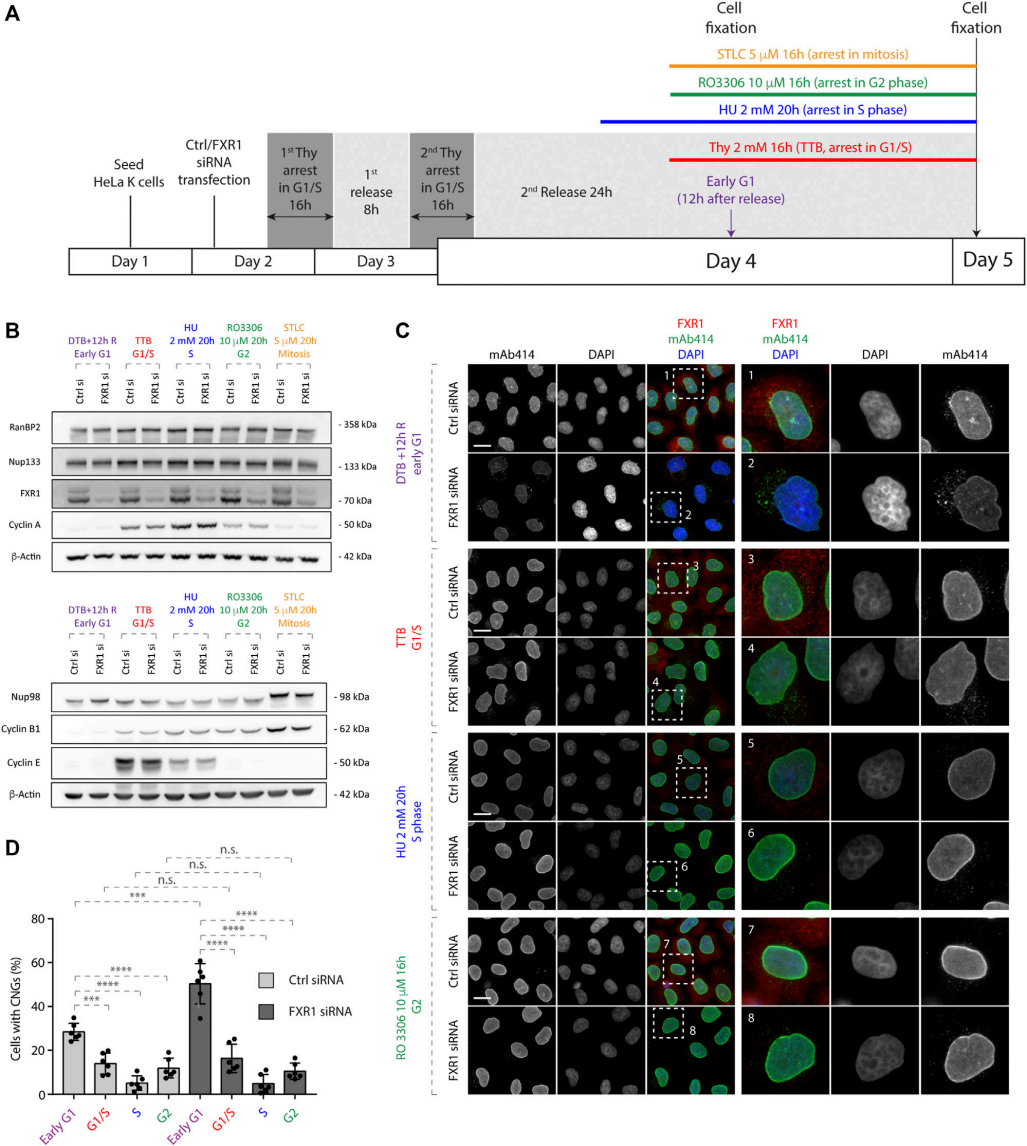
Nup localization is regulated by FXR1 in a cell cycle–specific manner. **(A)** Scheme of the experimental setup. HeLa K cells were seeded on coverslips, treated with the indicated siRNAs, synchronized in different cell cycle stages by different treatments: double thymidine block, and released for 12 h (DTB+12 h R, early G1); triple thymidine block (TTB, arrest in G1/S transition); incubation in 2 mM hydroxyurea (HU) for 20 h (arrest in the S phase); incubation in 10 µM RO3306 (CDK1 inhibitor) for 16 h (arrest in the G2 phase); and incubation in 5 µM STLC for 16 h (arrest in early mitosis). Subsequently, the cells were analyzed by Western blotting **(B)** and immunofluorescence microscopy **(C, D)**. **(B)** Whole cell lysates from cells treated as in **(A)** were analyzed by Western blot. **(C, D)** Cells treated as in **(A)** were analyzed by immunofluorescence microscopy **(C)** and the percentage of cells with cytoplasmic nucleoporin granules (CNGs) was quantified. **(D)** 9,800 cells were analyzed (mean ± SD, ***p < 0.001; ****p < 0.0001; n.s. = non-significant, N = 6). The magnified framed regions are shown in the corresponding numbered panels. Data information: scale bars are 5 μm. Statistical significance was assessed by one-way ANOVA test with Sidak’s correction.

To test if the regulation of Nup localization by FXR1 is coupled to cell cycle progression, we synchronized the control and FXR1-deficient HeLa cells in different cell cycle stages using several treatments: DTBR for 12 h for early G1, triple thymidine block for G1/S transition, hydroxyurea for the S phase, CDK1 inhibitor RO3306 for G2, and STLC for mitosis (Figure 3A). As expected, several FXR1 isoform protein levels were significantly decreased upon FXR1 siRNA treatment relative to the non-targeting control siRNA treatment (Figure 3B). Western blotting with antibodies to several cell cycle markers confirmed efficient synchronization of cells where cyclin E was accumulated during G1/S transition and decreased along the S phase, cyclin A levels gradually increased peaking in the S phase, and cyclin B1 gradually increased reaching the highest concentration in mitosis (Figure 3B). None or very moderate differences in the protein levels of Nup133 and RanBP2 were observed during different cell cycle stages in the control and FXR1-deficient cells, while Nup98, which is known to be phosphorylated during mitosis (Laurell et al., 2011), displayed an upshifted increased signal in STLC-treated cells. Interestingly, the downregulation of FXR1 led to significant increase of cells with CNGs solely in the early G1 phase but not in other cell cycle stages relative to the control-depleted cells (Figures 3C,D). Moreover, the CNGs observed in the control cells during early G1 decreased significantly during the following cell cycle stages in a pattern similar to FXR1-deficient cells (Figures 3C,D), suggesting that the tendency to form granules by the cytoplasmic Nups during early G1 is a physiological process.

**FIGURE 4:**
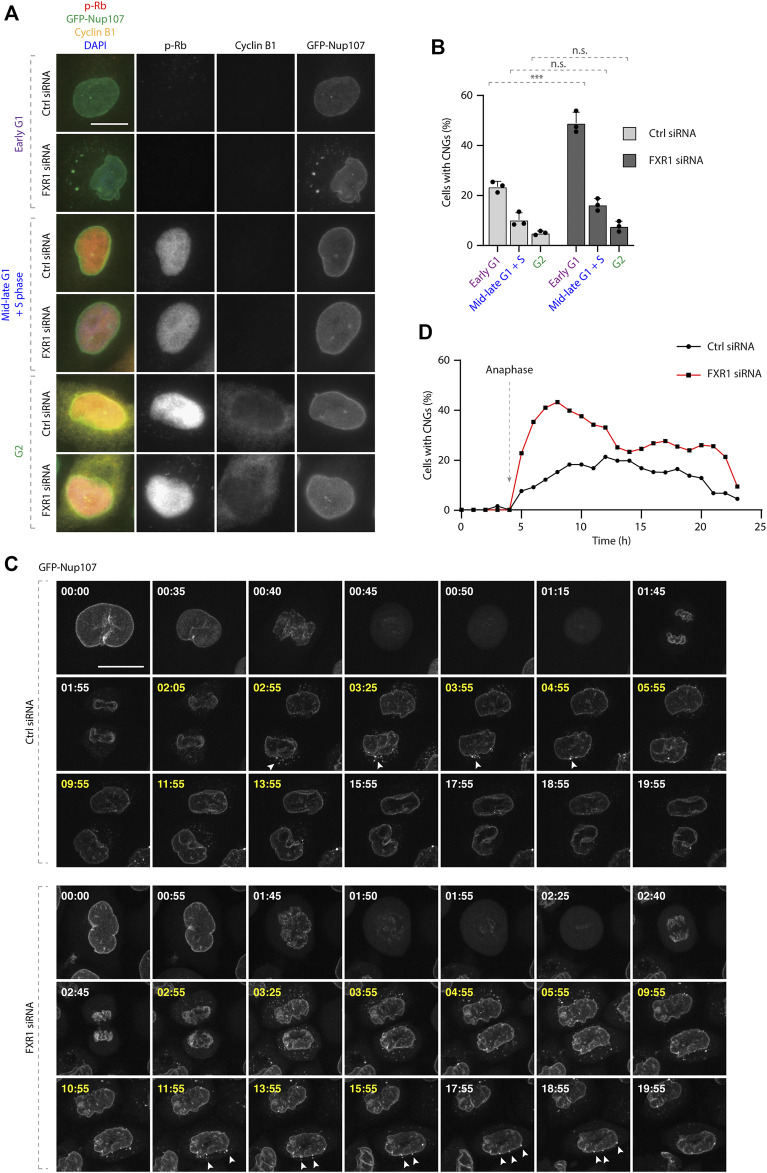
CNGs are present specifically during the G1 phase. **(A**–**B)** Asynchronously proliferating HeLa cells stably expressing GFP-Nup107 were treated with indicated siRNAs and analyzed by immunofluorescence microcopy **(A)** and the percentage of cells with cytoplasmic nucleoporin granules (CNGs) was quantified in different cell cycle stages. **(B)** 2,022 cells were analyzed (mean ± SD, ***p < 0.001; n.s. = non-significant, N = 3). **(C–D)** HeLa cells stably expressing GFP-Nup107 were treated with indicated siRNAs, synchronized by double thymidine block, and released and analyzed by live video spinning disk confocal microscopy starting 8 h after release and for 24 h. **(C)** Selected frames of the corresponding movies (Supplementary Movie S1 and S2) are depicted, and time is shown in hours. Time frames marked with yellow indicate the presence of CNGs. White arrowheads point to the cytoplasmic GFP-Nup107 granules fusing with the NE. The percentage of cells with cytoplasmic GFP-Nup107 granules over time was quantified in **(D)**. 154 cells were analyzed, and the onset of anaphase was artificially aligned at 4 h for all movies; scale bars are 5 μm.

To corroborate these findings in non-treated, asynchronously growing cells, we analyzed the localization of the retinoblastoma (Rb) protein which is phosphorylated during G1/S phase transition (p-Rb) and cyclin B (G2 marker) in cells stably expressing GFP-Nup107. The downregulation of FXR1 led to significant increase of cells with CNGs solely in the early G1 phase (p-Rb-negative) but not in cells in mid-late G1 and S phases (p-Rb-positive) or in G2 cells (p-Rb and cyclin B positive) (Figures 4A,B).

Live video spinning disc microscopy of cell lines stably expressing GFP-Nup107 and synchronized by DTBR demonstrated that GFP-Nup107–positive CNGs were first detected approximately 1 h after anaphase onset both in the control and FXR1-depleted cells (Figures 4C,D, Supplementary Movies S1–3). The number of CNG-containing cells was increased in FXR1-depleted cells relative to the control cells, but in both experimental conditions, CNGs were gradually attached to the NE in the time period of 11–16 h after anaphase onset (Figures 4C,D, Supplementary Movies S1–3). Considering that the peak of synchronized G1 cell population occurs at 11 h and the S phase peak at 16 h in a typical duration of HeLa cell cycle of 22 h (Posakony et al., 1977), our results indicate that CNGs disappear or are not formed as cells complete the G1 phase.

Collectively, our results show that the regulation of Nup localization by FXR1 is likely independent of translational processes and is restricted to a specific cell cycle stage. Moreover, autophagy and proteasome degradation mechanisms do not seem to be involved in the turnover of CNGs, raising a possibility that condensation-prone cytoplasmic Nups are incorporated into the NE during early G1.

## Discussion

### A Pool of Cytoplasmic Nups Is Regulated by FXR1 During Early G1

Our previous published results indicated that FXRPs and dynein-dependent microtubule transport can facilitate the dispersal of Nups and drive the localization of Nups to the NE, likely contributing to the function of NPCs in nuclear export (Agote-Aran et al., 2020). However, it was not clear if the FXRP-dynein pathway regulates a pool of nucleoporins remaining in the cytoplasm after postmitotic NPC assembly or being newly produced in early interphase. Although the protein levels of various Nups were unaffected by the depletion of FXRPs (Agote-Aran et al., 2020), it could not be excluded that the translation processes of yet to be identified Nups or Nup-associated factors are not involved in the FXRP-dependent Nup assembly. This is supported by the fact that translational regulation represents one of the best-studied roles of the FXR protein family (Darnell et al., 2009; Ascano et al., 2012). Interestingly, our results presented here argue against this possibility as the treatment with the translation inhibitors, namely, CHX and puromycin, did not affect the formation of CNGs induced by microtubule depolymerization and also did not lead to an increase in the formation of CNGs in the control cells (Figures 1C,D). It is very likely that under these experimental conditions, no new Nup proteins could be produced as indicated by a strong reduction in cyclin D levels (Figure 1B). This suggests that at the early G1 phase, the majority of existing cytoplasmic Nups was synthesized in the previous cell cycle and remained in the cytoplasm after the completion of the postmitotic NPC assembly pathway. In contrast to our findings, the ongoing translation of Nups was suggested to contribute to effective condensation into granules prior to AL assembly (Hampoelz et al., 2019). These differences could be explained by the rapid AL-NPC assembly process and quick cell divisions occurring in the oocytes, relative to slower cell cycle progression and the interphase NPC assembly process in cultured human cells. Interestingly, localized translation events of Nups have been linked to the biogenesis of NPCs in yeast (Rouvière et al., 2018; Lautier et al., 2021). Thus, although our data do not support the role of protein translation in the formation of CNGs at this point, we can still not formally exclude that the biogenesis of functional NPCs at the NE during G1 is not dependent on the localized protein translation of Nups or associated factors. The use of super-resolution microscopy analysis and quantifications of fully assembled NPC numbers upon the inhibition of protein translation should address this issue in the future.

Nevertheless, based on our new data, we hypothesize that a pool of Nups in the cytoplasm exists in early G1 and allows for a smooth transition from the postmitotic NPC assembly pathway to the interphasic pathway without relying on the global changes in the translation of Nups. This idea is in line with the fact that the RanBP2 and Nup133 protein levels are not affected by translation inhibition during early G1 phase (Figure 1D). This pool of Nups might be prone to condensation likely due to the absence of their incorporation into the growing nucleus. Remarkably, the CNGs observed in the control and in FXR1-deficient cells during early G1 tend to disappear as the cells progress to the next cell cycle, and both lysosomal and proteasomal degradation pathways appear not to be involved in the turnover of these Nup granules (Figure 2). In the future, it will be important to identify and characterize novel factors that besides FXRPs and dynein can lead to the incorporation of Nups to the NE during the early G1 phase.

### Why Are the Cytoplasmic Nups Regulated by FXR1 During Early G1?

Our new results suggest that CNGs can be specifically formed during the early G1 phase under both physiological conditions and at an increased rate upon the depletion of FXR1, and a smaller number of cells with Nup granules could be found beyond G1/S transition (Figures 3C,D, Figures 4A–D). Moreover, CNGs appear to dissolve and to be incorporated into the NE during the G1 phase (Figures 4C,D, Supplementary Movies S1–3). Why are the cytoplasmic Nups regulated by the FXRPs specifically during early G1? And what is the interplay of this novel pathway with the established NPC assembly mechanisms that act in the postmitotic and interphasic cells? A burst of NPC biogenesis has been observed immediately after NE reformation (Dultz and Ellenberg, 2010; Rampello et al., 2020). Given the small time window in which this increased NPC biogenesis may take place and the absence of major changes in the Nup protein levels at this time, both in the presence and absence of FXRPs (Agote-Aran et al., 2020) (Figure 1B), it is reasonable to think that the Nups remain in the cytoplasm to feed this wave of NPC assembly at the transition from mitosis to the early G1 phase. We therefore speculate that the FXRP-dependent pathway may, at least partially, contribute to the assembly of the functional NPCs at the NE during the early G1 phase as a part of the interphasic NPC assembly pathway. In future, super-resolution and electron microscopy analyses of NE-embedded NPCs should determine precisely any possible changes in the numbers of functional NPCs upon the inactivation of FXRP pathway components. Likewise, the molecular composition of CNGs should be studied in detail to understand if other proteins and/or RNAs can be sequestered to these cellular assemblies, and if they represent AL or AL-like structures, which have been recently implicated in NPC assembly as an intermediate step in the postmitotic pathway in higher eukaryotic cells (Ren et al., 2019). Additionally, correlative light and electron microscopy (CLEM) experiments could also address this issue and provide structural information on CNGs. It will be also important to determine if the FXRP–Nup pathway can be exploited upon changing the cellular conditions. Indeed, the number of NPCs can be modulated in response to cellular needs, for instance, during differentiation processes when the number of NPCs and nucleocytoplasmic trafficking dramatically increase (Kau et al., 2004). Furthermore, several Nups are involved in the differentiation processes into muscle and neuronal lineages (D’Angelo et al., 2012; Gomez-Cavazos and Hetzer, 2015; Jacinto et al., 2015). Future studies are required to address the physiological relevance of the FXRP-dependent regulation of NPC homeostasis during early G1 and the consequences of the misregulation of this pathway for human diseases.

## Materials and Methods

### Cell Lines, Cell Cycle Synchronizations, and Treatments

Human retinal pigment epithelial-1 (RPE-1) cells were cultured in Dulbecco’s modified Eagle medium (DMEM) F-12: 2.5 mM L-glutamine, 15 mM HEPES, 0.5 mM sodium pyruvate, and 1,200 mg/L sodium bicarbonate supplemented with 10% FCS, 0.01 mg/ml hygromycin B, and DLD-1 (colorectal adenocarcinoma epithelial cells). The cell line was maintained in RPMI-1640 medium: 2 mM L-glutamine, 10 mM HEPES, 1 mM sodium pyruvate, 4,500 mg/L glucose, and 1,500 mg/L sodium bicarbonate supplemented with 10% FCS and gentamicin.

The HeLa Kyoto cell line and derived stable cell line GFP-Nup107 (purchased from CSL cell bank) were cultured in Dulbecco’s modified Eagle medium (DMEM) (4.5 g/L glucose, with GLUTAMAX-I) supplemented with 10% FCS, 1% penicillin, and 1% streptomycin. The cells were synchronized by the addition of thymidine twice (Sigma, T1895) at 2 mM for 16 h. The cells were washed after each thymidine addition three times with warm medium to allow for synchronous progression through the cell cycle. The cells were treated and analyzed at desired time points after the release from the second thymidine block. Alternatively, the cells were synchronized in G1/S transition by three times addition of thymidine at 2 mM for 16 h (triple thymidine block, TTB). The cells were synchronized in the S phase by incubation in 2 mM hydroxyurea (HU, Sigma H8627-1G) for 20 h. The cells were synchronized in the G2 phase by incubation in 10 μM RO3306 (Cdk1 Inhibitor IV, Calbiochem 217699) for 16 h and in mitosis by the addition of 5 µM S-trityl-L-cysteine (STLC) (Eg5 inhibitor, Enzo Life Sciences, Ref. ALX-105-011-M500) for 16 h.

To inhibit translation, the cells were incubated with the translational inhibitors [100 μg/ml cycloheximide (CHX, Sigma C4859) or 200 μg/ml puromycin (Life Technologies, A11138-03)] or the vehicles (DMSO 1:1,000 and HEPES KOH 200 nM) in culture media for 7 h at 37°C.

To induce the formation of the cytoplasmic Nup granules by microtubule depolymerization, the cells were incubated with 10 μM nocodazole (Sigma M1404-50MG) in culture media for 90 min at 37°C.

To inhibit proteasomal degradation and/or autophagy, the cells were incubated with 10 μM MG132 (proteasome inhibitor, Sigma C2211) and/or 30 nM bafilomycin (Sigma, B1793-10UG), respectively, or the vehicle (DMSO, 1:10,000 or 1:500, respectively) in culture media for 12 h at 37°C.

To inhibit lysosomal hydrolases, the cells were incubated with 10 μg/ml of pepstatin A (Sigma P5318) and 10 μg/ml E64d (Sigma E8640) or the vehicle (DMSO, 1:100) in culture media for 4 h at 37°C.

### Immunofluorescence Microscopy and Sample Preparation

The cells were plated on 11-mm glass coverslips (Menzel-Glaser) in 24-well tissue culture plates. For Nup staining, at the end of the experiments, the cells were washed twice with PBS and fixed for 10 min with 1% PFA in PBS at RT. The coverslips were rinsed twice with PBS and permeabilized with 0.1% Triton X-100 and 0.02% SDS in PBS for 5 min at RT, washed twice with PBS, and blocked by the blocking buffer 3% BSA/PBS-T (0.01% Triton X-100) overnight. The coverslips were subsequently incubated with primary antibodies in blocking buffer for 1 h at RT, washed thrice for 5 min with blocking buffer, and incubated with secondary antibodies in blocking buffer for 30 min at RT in the dark. After incubation, the coverslips were washed thrice for 5 min with blocking buffer, then incubated in 0.1% Triton and 0.02% SDS in PBS for 1 min, and post-fixed in 1% PFA for 10 min. Finally, the coverslips were washed in PBS for 5 min and mounted on glass slides using Mowiol (Calbiochem) with 0.75 μg/μl DAPI and imaged with a 63X objective using a Zeiss epifluorescence microscope.

For lysosome staining with the Lamp1 antibody, at the end of the experiments, the cells were washed with PBS and fixed for 15 min with 4% PFA in PBS at RT. The coverslips were washed for 5 min with PBS thrice and permeabilized/blocked with blocking buffer (0.1% saponin, 3% BSA in PBS) for 1 h at RT. The coverslips were subsequently incubated with primary antibodies in blocking buffer for 1 h at RT and washed for 5 min with PBS thrice in 0.1% saponin in PBS. Then, the coverslips were incubated with secondary antibodies in blocking buffer for 1 h at RT in the dark. After incubation, the coverslips were washed for 5 min with PBS thrice and mounted on glass slides using Mowiol (Calbiochem) with 0.75 μg/μl DAPI and imaged with a 63X objective using the Zeiss epifluorescence microscope.

### Live Video Microscopy and Image Analysis

HeLa cells stably expressing GFP-Nup107 were treated with indicated siRNAs, synchronized by double thymidine block, released for 8 h, and analyzed by live video spinning disk confocal microscopy (Spinning Disk CSU-X1 “Nikon”) for 24 h. Z-stacks (10 μm range, 1 μm step) were acquired every 5 min, and movies were made with maximum intensity projection images for every time point shown at a speed of seven frames per second.

Image quantification analysis was performed using ImageJ software. Quantifications of the percentage of cells with cytoplasmic Nup granules were carried out by the eye.

### Experimental Design, Data Acquisition, Analysis, and Statistics

At least three independent biological replicates were performed for each experiment, except for the live video experiment which was performed once. The graphs were made using GraphPad Prism, Adobe Photoshop, and Adobe illustrator softwares.

All data were verified for normal distribution using the Shapiro–Wilk test. Normal data were analyzed using one-way ANOVA with Sidak’s correction for multiple group analysis. Error bars represent standard deviation (SD). In all cases, significance was *p < 0.05; **p < 0.01; ***p < 0.001; and ****p < 0.0001; n.s. = non-significant. The details for each graph are listed in the figure legends.

### siRNA Transfection

Oligofectamine (Invitrogen) was used to deliver siRNAs for gene knockdown according to the manufacturer’s instructions at a final concentration of 40–100 nM siRNA. The following siRNA oligonucleotides were used: non-silencing control siGENOME, non-targeting individual siRNA-2 5′-UAA​GGC​UAU​GAA​GAG​AUA​C-3′ (Dharmacon), and FXR1 siRNA 5′-AAA​CGG​AAU​CUG​AGC​GUA​A-3′ (Dharmacon).

### Western Blotting

Whole HeLa cell extracts were prepared using 1X Laemmli SDS sample buffer. The cells were washed twice in 1X PBS and incubated in 1X Laemmli SDS sample buffer for 30 min on ice. Subsequently, the samples were boiled at 96°C for 15 min and subjected to SDS-PAGE using bis-tris 4–12% gradient gels (Thermo Fischer NP0301BOX; NP0302BOX; NP0303BOX) or tris-acetate 3–8% gradient gels (Thermo Fischer EA0375BOX; EA03752BOX; EA03755BOX).

The proteins were subsequently transferred from the gel to a PVDF membrane (Millipore IPFL00010) for immunoblotting. The membranes were blocked in 5% non-fat milk powder resuspended in TBS supplemented with 0.1% Tween 20 (TBS-T) for 1 h at RT or overnight at 4 C, followed by incubation with antibodies. The membranes were developed with Luminata Forte (Millipore WBLUF0500).

### Antibodies

The following antibodies were used:

Mouse α-tubulin (Sigma T5169, Western blot 1:10,000), mouse α-β-actin (Sigma A2228-100UL, Western blot 1:10,000), mouse monoclonal α-FXR1+2 (clone 2B12 from IGBMC, Western blot 1:1,000), rabbit α-FXR1 (Sigma HPA018246, immunofluorescence microscopy 1:800), mouse α-FXR1 (Millipore 03–176, immunofluorescence microscopy 1:800), mouse α-Nup133 (Santa Cruz sc-37673, Western blot 1:1,000), mouse α-FG-Nups (Abcam mAb414, immunofluorescence microscopy ab24609, 1:500), mouse cyclin B1 (Santa Cruz sc-245, clone GSN1, immunofluorescence microscopy 1:300, Western blot 1:2000), rabbit α-cyclin A (Santa Cruz sc-751, Western blot 1:1,000), rabbit α-cyclin D1 (Santa Cruz sc-718, Western blot 1:1,000), mouse cyclin E (Santa Cruz sc-247, Western blot 1:1,000), rabbit α-RanBP2 (Abcam ab64276, immunofluorescence microscopy 1:500, Western blot 1:1,000), rabbit α-Nup98 (Cell Signaling 2598S, Western blot 1:1,000), guinea pig α-p62 (Interchim GP62-C, immunofluorescence microscopy 1:500), rabbit α-p62 (Genetex GTX100685, Western blot 1:1,000), mouse α-ubiquitin P4D1 (Cell Signaling 3936, Western blot 1:1,000), and rabbit α-p-Rb (Cell Signaling 8516, immunofluorescence microscopy 1:1,600).

## Data Availability

The raw data supporting the conclusion of this article will be made available by the authors, without undue reservation.

## Supplementary Material

Supplementary Figure S1 CNGs are present in non-transferred and chromosomally stable cancer cells. **(A–D)** RPE-1 **(A, B)** and DLD-1 **(C, D)** were treated with indicated siRNAs and analyzed by immunofluorescence microscopy **(A, C),** and the percentage of cells with cytoplasmic nucleoporin granules (CNGs) was quantified. **(B, D)** 1962 RPE-1 and 1713 DLD-1 cells were analyzed (mean ± SD, ***p* < 0.01; *****p* < 0.0001; n.s. = non-significant, N = 3).

Supplementary Figure S2 CNGs do not co-localize with the lysosomal marker Lamp1. **(A)** Scheme of the experimental setup. HeLa K cells were seeded on coverslips, treated with the indicated siRNAs, synchronized by double thymidine block, and released from G1/S arrest. Eight hours after release, the cells were incubated with lysosomal hydrolase inhibitors (pepstatin A 10 µg/ml and E64 10 µg/mL) or the vehicle (DMSO 1:100) for additional 4 h. Subsequently, the cells were prepared for lysosome staining with the Lamp1 antibody and analyzed by immunofluorescence microscopy **(B)**. **(B)** Cells treated as in **(A)** were analyzed by immunofluorescence microscopy. The magnified framed regions are shown in the corresponding numbered panels. Scale bar is 5 μm.

Supplementary Movie S1 CNGs are present specifically during the G1 phase. HeLa cells stably expressing GFP-Nup107 were treated with control siRNA, synchronized by double thymidine block, released, and analyzed by live video spinning disk confocal microscopy starting 8 h after release and for 24 h. The selected frames are shown in Figure 4C.

Supplementary Movie S2 CNGs are present specifically during the G1 phase. HeLa cells stably expressing GFP-Nup107 were treated with FXR1 siRNA, synchronized by double thymidine block, released, and analyzed by live video spinning disk confocal microscopy starting 8 h after release and for 24 h. The selected frames are shown in Figure 4C.

Supplementary Movie S3 CNGs are present specifically during G1 phase. HeLa cells stably expressing GFP-Nup107 were treated with FXR1 siRNA, synchronized by double thymidine block, released, and analyzed by live video spinning disk confocal microscopy starting 8 h after release and for 24 h.
