# Supplementary figures and images for: Fragile X–Related Protein 1 Regulates Nucleoporin Localization in a Cell Cycle–Dependent Manner
Source: Front Cell Dev Biol. 2021 Dec 16;9:755847. doi: 10.3389/fcell.2021.755847 (PMC8716781; doi:10.3389/fcell.2021.755847)

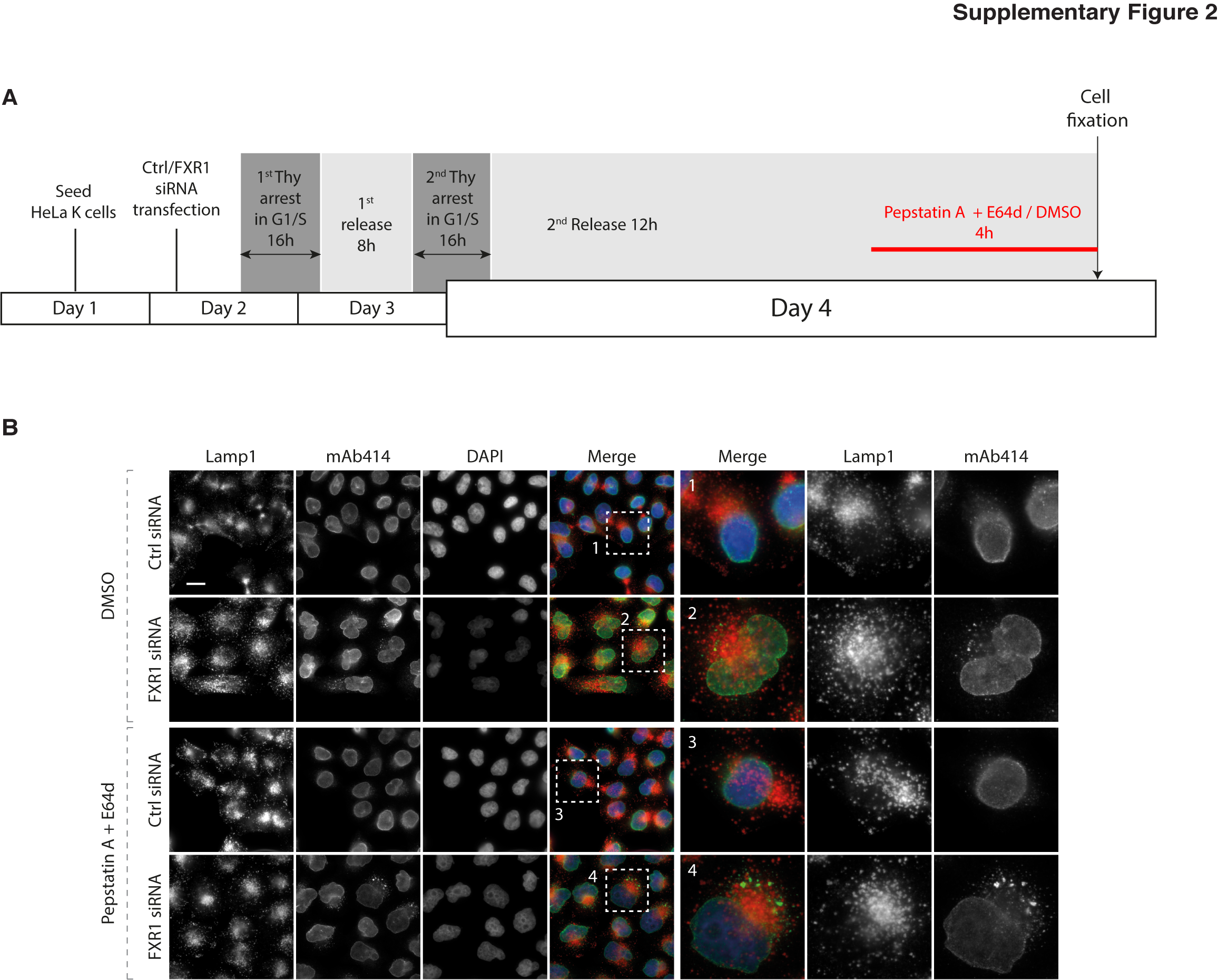

Supplement: Supplementary file 3 [file Image2.tif]

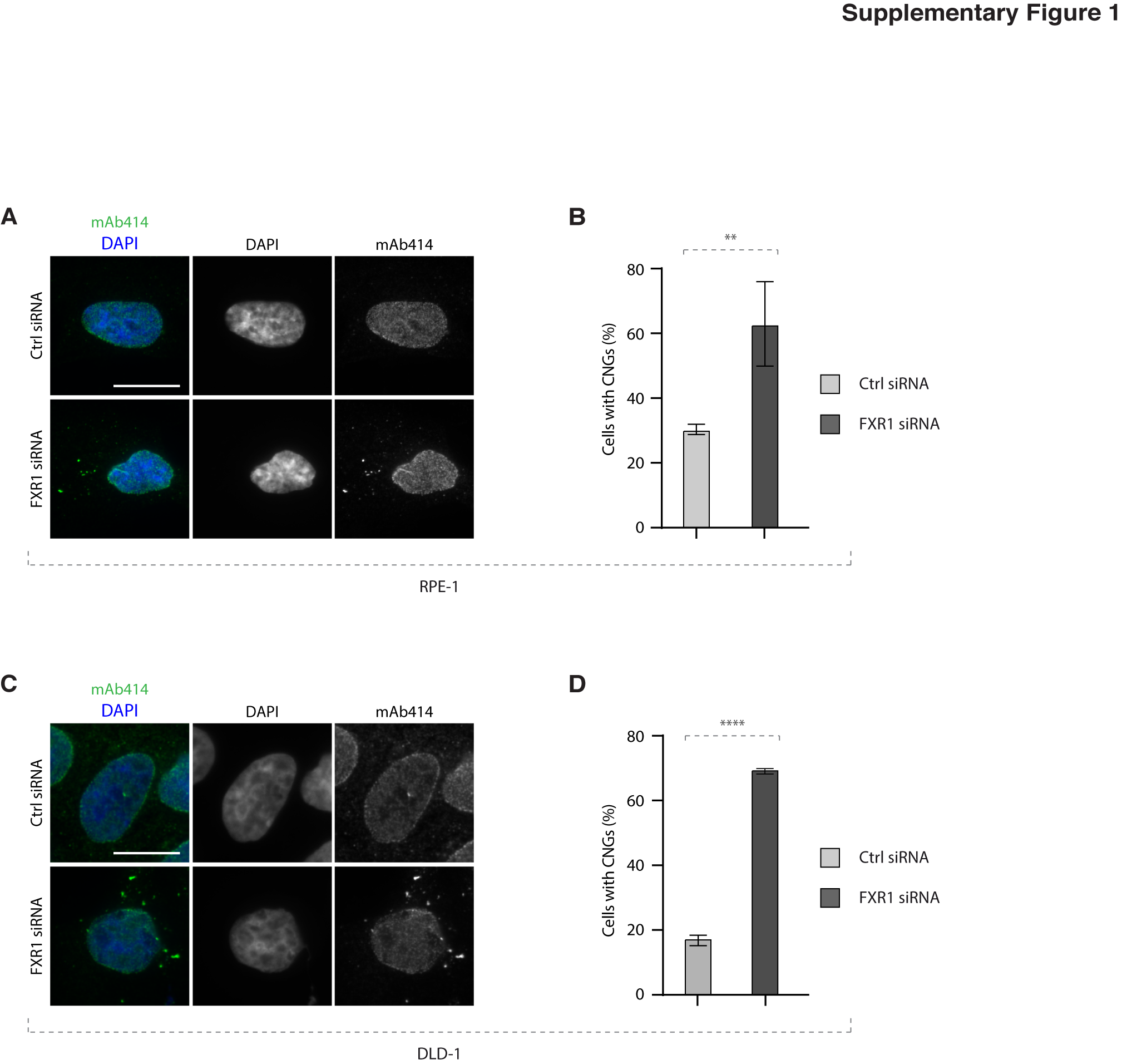

Supplement: Supplementary file 4 [file Image1.tif]
